# Memory deficits in Sprague Dawley rats with spontaneous ventriculomegaly

**DOI:** 10.1002/brb3.1711

**Published:** 2020-06-25

**Authors:** Hiram Luna‐Munguia, Deisy Gasca‐Martinez, Luis Marquez‐Bravo, Luis Concha

**Affiliations:** ^1^ Departamento de Neurobiologia Conductual y Cognitiva Instituto de Neurobiologia Universidad Nacional Autonoma de Mexico, Campus UNAM-Juriquilla Queretaro Mexico; ^2^ Unidad de Analisis Conductual Instituto de Neurobiologia Universidad Nacional Autonoma de Mexico, Campus UNAM-Juriquilla Queretaro Mexico

**Keywords:** decision making, hippocampus, magnetic resonance imaging, memory, ventriculomegaly

## Abstract

**Introduction:**

Spontaneous ventriculomegaly has been observed in rats that were presumed normal. Because the external phenotype of these animals is unremarkable, they can be inadvertently included in behavioral experiments, despite the considerable enlargement of the ventricular system, reduced cortical thickness, and hippocampal atrophy upon imaging. Given the role of such structures in memory consolidation, we evaluated long‐term memory retention while decision making in rats with spontaneous ventriculomegaly.

**Methods:**

We studied adult male Sprague Dawley rats, identified as having spontaneous ventriculomegaly, while performing baseline magnetic resonance imaging scanning intended for a different research protocol. Control (*n* = 7) and experimental (*n* = 6) animals were submitted to a delayed‐alternation task (no delay, 30, 60, and 180 s) and an object‐in‐context recognition task. During the first task, we evaluated the number of correct choices as well as the latency to reach any of the cavities located at the end of each branch arm during each trial. The second task assessed the rodents’ ability to remember where they had previously encountered a specific object, calculating the context recognition index.

**Results:**

When compared to control animals, rats with spontaneous ventriculomegaly required significantly more training sessions to reach the 80% criterion during the training phase. Moreover, they showed reduced delayed‐alternation performance in the evaluated times, reaching significance only at 180 s. Increased latencies while trying to reach the cavity were also observed. Evaluation of the long‐term memory formation during the object‐in‐context recognition task showed that subjects with ventriculomegaly spent less time investigating the familiar object, resulting in a significantly decreased recognition index value.

**Conclusion:**

Our results are the first to show how spontaneous ventriculomegaly‐induced cerebral structural damage affects decision‐making behaviors, particularly when comparing between immediate and delayed trials. Moreover, this lesion disrupts the animals’ ability to recall or express contextual information.

## Graphical abstract

Spontaneous ventriculomegaly is a severe noninduced cerebral lesion. However, animals try to decode the challenges when exposed to memory tasks despite their grossly abnormal brain anatomy. This suggests the existence of compensation mechanisms.

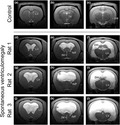

## 1 INTRODUCTION

Hydrocephalus is a multifactorial brain condition that usually occurs due to a dynamic imbalance between production and absorption of cerebrospinal fluid, resulting in a pathological dilation of the cerebral ventricles (Zhang, Williams, & Rigamonti, 2006; McAllister 2012). This complexity has led to several classification schemes based on diverse criteria. Hydrocephalus has been categorized as congenital (present at birth and often associated with developmental defects) or acquired (occurs after brain and ventricles development as a complication of another condition such as hemorrhage, infection, or neoplasm) (Mori, Shimada, Kurisaka, Sato, & Watanabe, 1995; Tully & Dobyns, 2014). To understand the underlying causes of hydrocephalus, several animal models have been developed, including rodents and zebrafish (Lopes, Slobodian, & del Bigio, 2009; McAllister 2012; Korzh, 2018).

Congenital spontaneous hydrocephalus has been previously reported in diverse experimental rat strains. While this condition is estimated to be extremely rare in mice (https://www.jax.org/news‐and‐insights/2003/july/hydrocephalus‐in‐laboratory‐mice), many other groups have reported it anecdotally in mice and rats (see a recent discussion at https://twitter.com/AdrianDu_/status/1181663402898407425). Tu et al. (2014, 2017) found mild spontaneous ventriculomegaly in 43% of Wistar rats that were presumably normal when sent from two different vendors, highlighting the lack of phenotypic and gross behavioral manifestations. Similarly, we noticed evident cerebral abnormalities in 15% of the Sprague Dawley rats obtained from our Institute's animal facility while conducting a longitudinal study that required baseline magnetic resonance imaging (MRI) scanning. Prior to imaging, there were no signs of cerebral abnormalities, as ventriculomegalic rats displayed similar skull size and body weight to those of normal rats. It has been described that if the ventricular enlargement is severe and the cerebrospinal fluid is not drained, the animals expire within the first weeks of life (Sasaki et al., 1983). Contrastingly, we found that these animals can indeed develop normally into adulthood and display an apparent normal behavior.

The hippocampus is a limbic system structure particularly important in forming new memories. Three of the primary roles of the hippocampus while acting as a memory buffer are as follows: (a) to create flexible representations of context and object associations (Spanswick & Sutherland, 2010), (b) to impact upon decision‐making behaviors (Gleichgerrcht, Ibañez, Roca, Torralva, & Manes, 2010; Gupta et al., 2009), and (c) to consolidate long‐term memories (Floresco, Seamans, & Phillips, 1997; Hölscher, 2003). In human patients with ventriculomegaly, their declined memory formation is related to an atrophied hippocampal formation, which is not surprising considering that the hippocampus neighbors the lateral brain ventricles (Golomb et al., 1994; Peterson et al., 2018). Other studies have also related childhood hydrocephalus to cognitive disorders (Lindquist, Persson, Uvebrant, & Carlsson, 2008; Zielinska, Rajtar‐Zembaty, & Starowicz‐Filip, 2017), possibly attributable to the destruction of neural circuits and change of cerebral neurotransmitter levels (del Bigio, 2010; Engelsen, Fosse, Myrseth, & Fonnum, 1985).

The goal of this work is to evaluate memory performance in rats with spontaneous ventriculomegaly by subjecting them to a couple of tasks in which some of the altered/displaced brain regions are crucial for the animals to create long‐term retention memories while making decisions.

## 2 MATERIALS AND METHODS

### 2.1 Animals

Given the nature of our original research protocol, we worked with male and female rats. Spontaneous ventriculomegaly was found in 15% of our male group while only two females out of a group of 40 animals presented the severe ventricular enlargement. Females showed the same severe lesion but were not included in the memory tasks.

Thirty five‐day‐old male Sprague Dawley rats were individually housed in a facility under controlled illumination (12‐hr light/dark cycle; light on 07:00 a.m.) and environmental conditions (21 ± 1°C, 50%–60% humidity). Access to food and water ad libitum in their acrylic home cages. All animals were acclimatized to the room conditions for at least 5 days before any experimental manipulation. All procedures were carried out in accordance with protocols approved by our Institutional Ethics Committee for Animal Use and Care (CICUAL‐INB; Protocol #105A) and following the Official Mexican Standard regulations (NOM‐062‐ZOO‐1999).

### 2.2 Magnetic Resonance Imaging (MRI)

Forty‐day‐old animals were anesthetized with a 4% isoflurane/air mixture concentration for induction and positioned on the cradle while fixing the subject's snout to the bite bar. A 2% mixture concentration was used to maintain anesthesia during image acquisition. Body temperature (37°C) was preserved using a warm water circulation system beneath the rat. Respiration rate and oxygen saturation (40–60 breaths/min and 85%–90%, respectively) were continuously monitored. Anesthesia was discontinued upon termination of the imaging session. Animals were closely observed until full recovery and transferred to the housing room.

MRI acquisition protocols were conducted with a 7 Tesla MRI scanner (Bruker Pharmascan 70/16US) interfaced to a Paravision 6.0.1 console, using a 2 × 2 surface array coil. A 3‐min anatomical scan was acquired with the following parameters: repetition time (TR) = 350 ms, echo time (TE) = 2.4 ms, number of averages (NA) = 3, flip angle = 30°, field of view (FOV) = 25 × 18 mm^2^, matrix = 250 × 180 (yielding 100 µm in‐plane resolution), slice thickness = 800 µm, and number of slices = 20. This imaging sequence was part of a different research project. Animals that displayed the characteristic features of ventriculomegaly were discarded from that study and included in this work. A second MRI scan was performed under the same parameters one week apart from the last memory task session to evaluate time‐dependent changes.

Volumes of the lateral ventricles were assessed by manual delineation on the MRI images obtained from both scans using ITK‐Snap (Yushkevich et al., 2006). The rostral and caudal margins of the *corpus callosum* served as anatomical landmarks to limit segmentation.

### 2.3 Behavioral evaluation

All behavioral tests were performed in the Behavioral Analysis Unit of the Institute of Neurobiology. A specific room was used for each task. Rooms had controlled environmental conditions (22 ± 1°C, 50%–60% humidity).

#### 2.3.1 Open field test

Locomotor activity was evaluated before submitting the animals to the cognitive tasks. For five consecutive days, rats were placed in an open‐field arena (42 *L* × 42 *W* × 39 *H *cm) for 10 min. A video camera positioned above the arena recorded the motor activity of all the animals. Videos were analyzed using the SMART 3.0 Vision software (Pan Lab Harvard Apparatus) by an observer blind to allocations.

#### 2.3.2 Delayed‐alternation task

The aim of this task was to evaluate the animals’ decision making based on the alternation behavior rather than the decision making based on the achievement of the reward.

**FIGURE 1:**
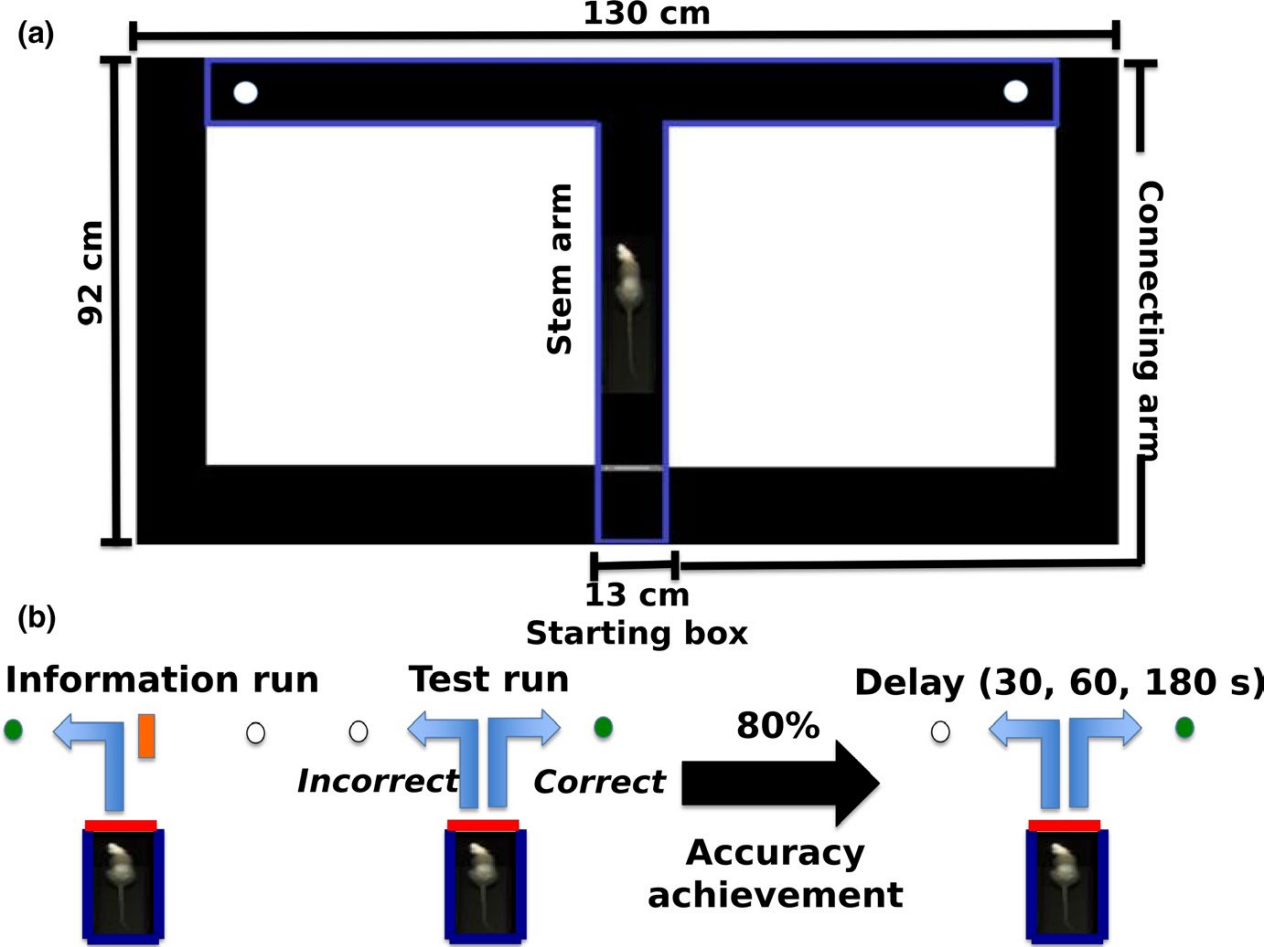
The modified T‐maze apparatus and experimental procedure. (a) The illustration shows the modified T‐maze apparatus. (b) The scheme shows the experimental procedure for performing the delayed‐alternation task in the modified T‐maze

In this study, ten days after the first MRI scan, control animals (*n* = 7) and rats with ventriculomegaly (*n* = 6) were submitted to the delayed‐alternation task using a modified T‐maze made of black Plexiglas runways (Figure 1a). This structure has a central runway or stem arm (92 length (*L*) × 13 width (*W*) × 10 height (*H*) cm), a crosspiece that integrates both branch arms (130 *L* × 13 *W* × 10 *H *cm), additional runways connecting the distal ends of the branch arms to the stem runway (157 *L* × 13 *W* × 10 *H *cm), and two small circular cavities (radius = 1 cm and depth = 0.5 cm) located on the floor at the end of each branch arm. The maze is located at the center of the testing room and elevated 60 cm above the floor level.

Water was always available in the home cage while chow allowance was kept at 90% of the normal intake throughout the experiment in order to increase the animals’ food motivation (Mizoguchi et al., 2000). Five days before starting the training session, each rat was manipulated and habituated to the modified T‐maze (10 min/day), receiving ~4 g of food/day as a delivered reward inside the structure during this process. After habituation, the animals were trained on the delayed‐alternation task (Figure 1b). During the first trial (information run), the rat was placed in the starting box of the stem arm and one branch arm of the maze was blocked, forcing the subject to go to the open branch arm, where the animal was rewarded with one chocolate‐flavored whole grain cereal. The rat was prevented from retracing the route of the chosen arm, inducing its walk along the connecting arm in order to return to the initial point (starting box), which was blocked upon entry. The second trial (test run) started immediately afterward. For this, the starting box was opened and the animal was allowed to run down along the stem arm and freely choose from either branch arms. Animals picking the previously nonvisited arm (correct choice) received a reward, whereas those re‐visiting the previous arm were not rewarded. Once the rat made its choice, the entrance to the elected route was blocked and the subject was induced to return to the starting box. Left or right allocations for both the information and the test run were randomized over 10 different trials per day, with no more than three consecutive information runs done on the same side (Fellows, 1967; Wang, Liu, Hung, & Chen, 2014). Regardless of the number of sessions required, once the animal reached a rate of 80% correct choices, different delay times (no delay, 30, 60, and 180 s) between the information and the run test were evaluated. All rats were submitted once to each delayed time per day based on the working sequence previously described. Animals had to wait in the fully blocked starting box between each delayed‐time trial. The T‐maze was wiped down with 70% ethanol solution to reduce odor cues between trials. An arm choice was defined when the four paws of the subject were rested within one of the T‐maze arms. We recorded and evaluated the number of correct choices as well as the latency to find the cavity during the information and the test run.

#### 2.3.3 Object‐in‐context recognition task

To assess contextual recognition memory, we partially modified the method described by Balderas et al. (2008). Briefly, we used two arenas with different contexts, located in a quiet and brightly lit room. The first arena (context 1) was a black acrylic square (42 *L* × 42 *W* × 39 *H *cm), while the second one (context 2) was a black acrylic box with different measures (40 *L* × 60 *W* × 50 *H *cm) and visual features (two walls had black and white stripes, the other two had white circles). A video camera mounted 90 cm directly above the center of each box was used to record each session.

One week after being submitted to the delayed‐alternation test, all animals from both groups (*n* = 13) were taken to the object‐in‐context recognition task. Here, for five consecutive days, the rats were handled for a minute. Immediately afterward, each subject was habituated to context 1 (without any object) for 3 and 90 min later to context 2 under the same conditions. The sixth day was designated for sample phase 1, where the animals were allowed to freely explore context 1 with two different objects inside (A1 and B1) for 10 min. Sample phase 2 was conducted 24 hr later, placing each rat in context 2 and exposing it to a couple of copies of one of the previously presented objects (A2 and A3) for 10 min. At this point, we consider that contexts and objects are familiar to the animals. Therefore, long‐term memory was tested 24 hr later. For this, all subjects were placed again into context 2 for 3 min, allowing them to explore two different objects: a copy of the previously presented object in context 2 (A4) and a copy of the object previously presented in context 1 but not presented in context 2 (B2). Context recognition indexes were calculated as follows: time of exploration of familiar object in novel context/(time of exploration of familiar object in familiar context + time of exploration of familiar object in novel context).

### 2.4 Brain extraction

Two days following the second MRI scan, rats were deeply anesthetized using isoflurane inhalation and overdosed using an intraperitoneal injection of pentobarbital. Animals were transcardially perfused with 0.9% saline solution followed by a 4% paraformaldehyde solution. Brains were carefully removed and stored at room temperature in 4% paraformaldehyde solution. Qualitative anatomical evaluations were performed by sectioning the specimens coronally at the level of the ventral hippocampi.

### 2.5 Statistical analysis

Statistical analysis was conducted using GraphPad Prism8. Values were expressed as mean ± *SEM*. The number of days required to reach the 80% criterion on two consecutive training days and the latencies to find the cavity during the information run and test run were analyzed using a two‐way ANOVA composed of within (time) and between (groups) factors. For delayed‐rewarded alternation, we used a two‐way ANOVA followed by a *post hoc* Tukey test to compare each delayed‐time‐point between days 13 and 17. Based on previous studies (Balderas et al., 2008; Dix & Aggleton, 1999; Mumby, Gaskin, Glenn, Schramek, & Lehmann, 2002), only the first minute recorded from the object‐in‐context recognition memory test was used for statistical analysis (novel object discrimination is more evident during this period). Data were analyzed using an unpaired *t* test. In all statistical comparisons, *p* < .05 was used as a criterion for significance.

## 3 RESULTS

Control and ventriculomegalic animals were always weighed before the MRI scans and the day before starting each of the tasks. No body growth differences between groups were detected. Therefore, we suggest that basic functions (such as eating or drinking) of the animals with ventriculomegaly were not altered.

### 3.1 MRI‐detected spontaneous ventriculomegaly in male Sprague Dawley rats

**FIGURE 2:**
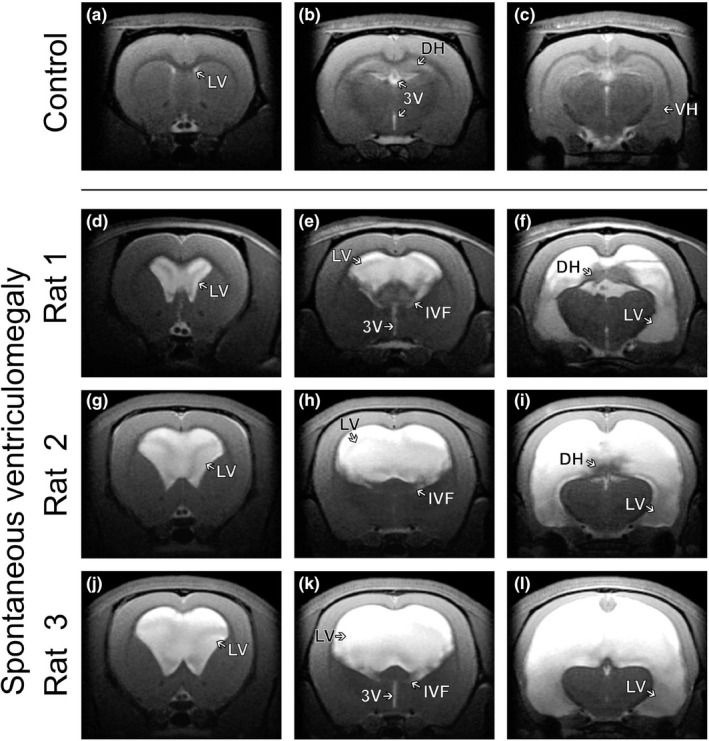
Control and spontaneous ventriculomegaly rat brains in anatomical T_2_‐weighted images acquired during the first MRI scan. (a–c) Coronal slices showing a rat with normal‐sized ventricles and limbic structures in a series of T_2_‐weighted images. (d–l) Coronal slices depicting different patterns and degrees of spontaneous ventriculomegaly in three different animals. (e, h, k) Communicating ventriculomegaly with bilateral enlargement of the lateral ventricles connected through the inter‐ventricular foramen to the 3rd ventricle. (f, i) Dorsal hippocampus retracted and compressed by the accumulating fluid in the lateral ventricles. (f, i, l) Ventral hippocampus completely covered and displaced by cerebrospinal fluid. 3V, 3rd ventricle; DH, dorsal hippocampus; IVF, inter‐ventricular foramen; LV, lateral ventricles; VH, ventral hippocampus

We found spontaneous ventriculomegaly in six animals out of a group of 40 animals that were scanned for other research purposes (i.e., 15%). Figure 2 shows the coronal slices from the first anatomical T_2_‐weighted MRI sequence (40‐day‐old animals). When comparing patterns between normal (Figure 2a–c) and lesion (Figure 2d–l) animals, considerably changed anatomy was observed. Quantitatively, ventricular volumes were greater in the experimental animals than the control subjects (353 mm^3^ ± 152 and 4.6 mm^3^ ± 0.9, respectively; *p* < .001). Each group showed no significant differences between the MRI values obtained from the first and the second scan.

### 3.2 Effect of spontaneous ventriculomegaly on locomotor activity

Ventriculomegalic rats showed normal motor abilities, as assessed by their overall displacement in an open‐field arena. No significant differences were observed between control and ventriculomegalic animals (total distance (cm) 7,867 ± 2,650 and 6,814 ± 2,236, respectively; *p* > .05).

### 3.3 Effect of spontaneous ventriculomegaly on delayed‐alternation performance

#### 3.3.1 Performance accuracy

**FIGURE 3:**
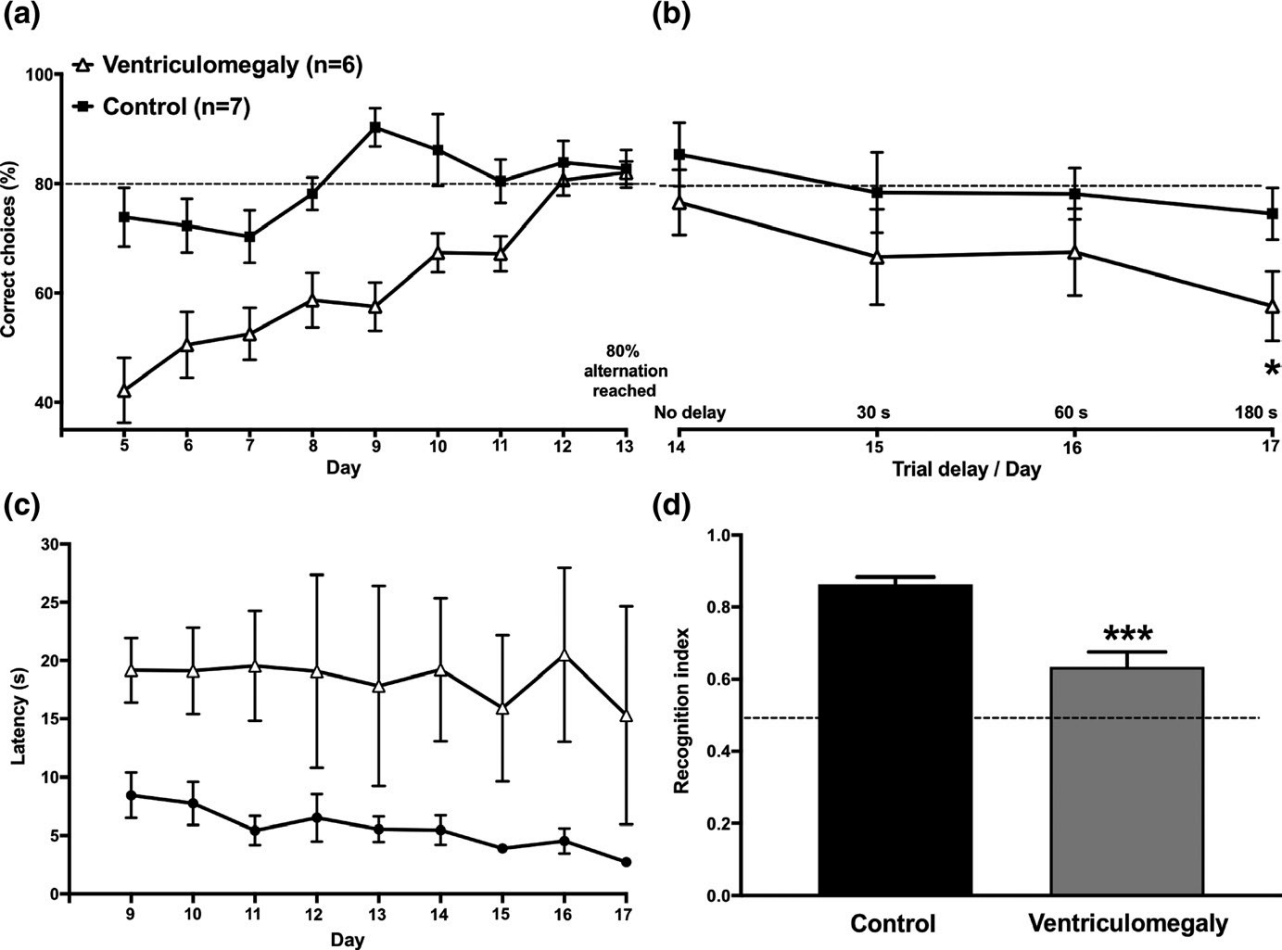
Performance accuracy of the tasks. (a) The percentage of correct choices was calculated to evaluate the performance accuracy of the T‐maze task. A two‐way ANOVA revealed differences between groups while trying to achieve the 80% alternation criterion. (b) The percentage of correct choices presented in the task with a delay of 15 s was gradually lost in both groups when extending the delay interval to 30, 60, and 180 s. Tukey's multiple comparisons showed that the effect was more pronounced in the ventriculomegalic animals; reaching significance when exposed to the 180 s delay. (c) Shows the latency to enter the goal arm during the Information Run. Rats with ventriculomegaly had greater values when compared to the control group. (d) Shows the recognition indexes on object recognition memory test. A Wilcoxon test shows a significant difference between groups. All data are expressed as mean ± *SEM*. *p < .05; ***p < .001

The percentage of correct choices was calculated to evaluate the performance accuracy in the modified T‐maze apparatus. Each point represents the average of the 10 trials per day. During the training phase, rats with spontaneous ventriculomegaly required significantly more training sessions to reach the 80% accuracy criterion than control animals (130 and 90 sessions, respectively) (Figure 3a). A two‐way ANOVA revealed significant differences while comparing the percentage of accuracy between groups and training days (*F*_1,11_ = 17.96, *p* = .001 and *F*_3.6,40.2_ = 12.83, *p* = .001, respectively). Magnitude (training day x group interaction) also differs across groups (*F*_8,88_ = 4.64, *p* = .001).

The second analysis was designed to evaluate the performance accuracy of the 13 animals that achieved the 80% accuracy criterion during the delayed‐spatial‐alternation test. A two‐way ANOVA revealed a significant main effect of the delay (*F*_3.43,41.12_ = 2.76; *p* = .047). No main effect on group or delay x group interaction was observed (*F*_1,12_ = 4.54; *p* = .054 and *F*_5,60_ = 0.577; *p* = .717, respectively). Tukey's multiple comparisons showed between‐group differences only at the delay interval of 180 s (*p* < .032) (Figure 3b).

#### 3.3.2 Latency

The latency to reach any of the two small circular cavities located on the floor at the end of each branch arm was also measured for each animal (*n* = 13) during the information and test run. Interestingly, and in contrast to control animals, rats with ventriculomegaly usually did not eat the food pellet despite being able to reach it; just sniffed around and continued their way through the connecting arm. A two‐way ANOVA showed that the lesion animals had significantly higher values during the information run (*F*_1,11_ = 9.625; *p* = .01). Figure 3c only shows values between days 9 and 17; statistics were done including only these days. No significant differences between groups were observed during the test run (*F*_1,12_ = 5.69; *p* = .35) (data not shown). Magnitude (training day x group interaction) did not differ across groups.

### 3.4 Effect of spontaneous ventriculomegaly on object‐in‐context recognition performance

Both groups spent similar time exploring each of the two objects during sample phase 1. Interestingly, on the long‐term memory probe, the rats with ventriculomegaly displayed an impaired ability to recognize familiar objects in novel contexts. Recognition index values shown in Figure 3d were compared between the control group (0.88 ± 0.02) and the lesion group (0.6 ± 0.03). An unpaired *t* test indicated a significant difference between groups (*p* < .001). This reveals that the ventriculomegaly group also showed long‐term context recognition memory impairment.

**FIGURE 4:**
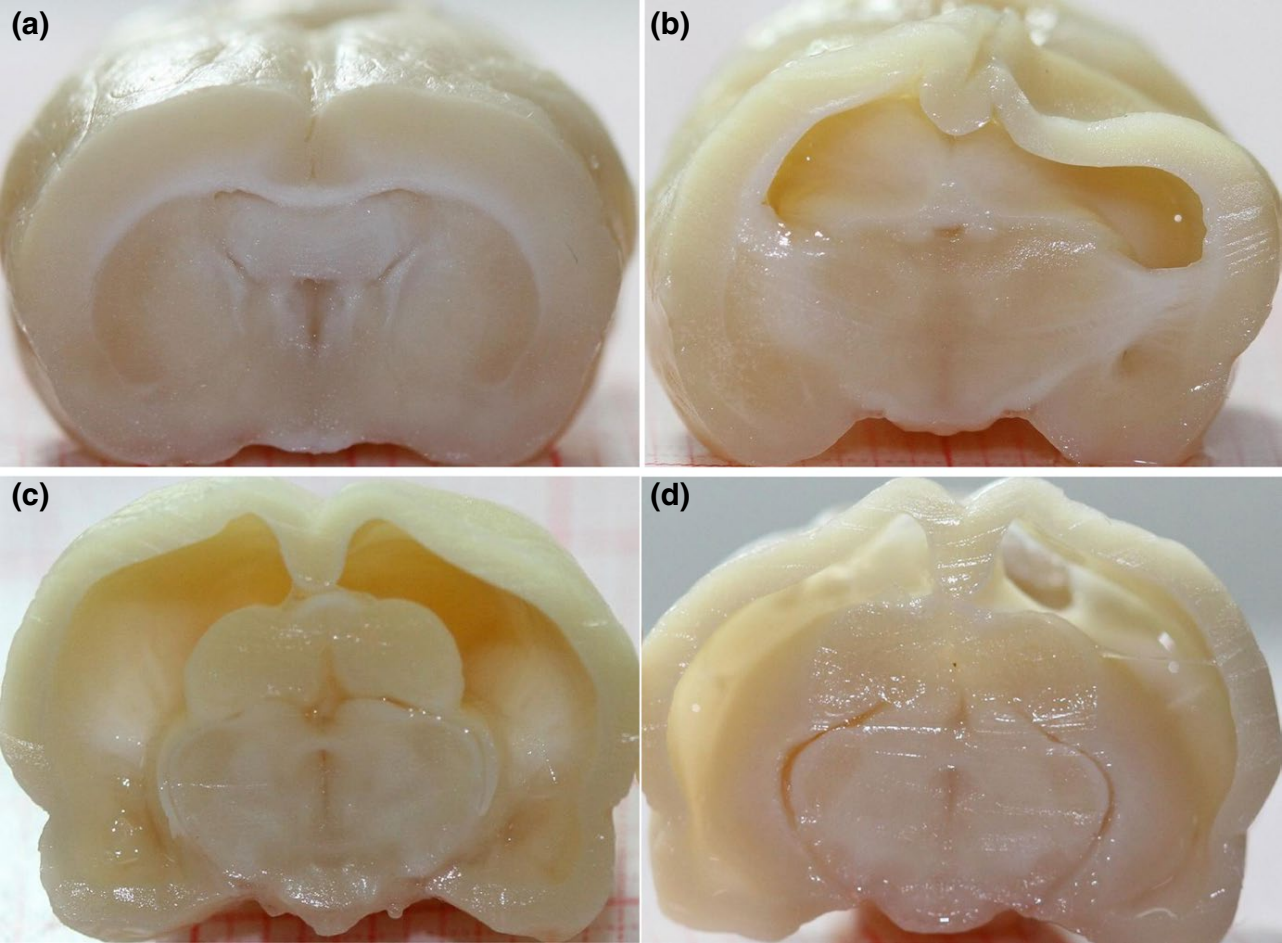
Brain structure. (a) Control animal. (b–d) Rats with ventriculomegaly show a very thin cerebral cortex and an enlarged ventricular system that displaces other brain regions

Examination of the excised brains (Figure 4) clearly showed that, in addition to the considerable enlargement of the ventricular system, ventriculomegalic animals had reduced cortical thickness and displaced or altered morphology of other brain regions such as caudate nucleus, *corpus callosum*, and limbic structures among others.

## 4 DISCUSSION

Animal models of neurological disorders are crucial for basic research. They have led to a better understanding of the cellular and molecular pathogenesis of several disorders such as Alzheimer's disease, Parkinson's disease, and epilepsy (Götz, Bodea, & Goedert, 2018; Metzger & Emborg, 2019; Nirwan, Vyas, & Vohora, 2018). In our case, while performing a baseline MRI scanning intended for a different research protocol, we noticed that 15% of the subjects had spontaneous ventriculomegaly despite their normal external appearance and apparent normal behavior. In consequence, we discarded these animals from the original project but were intrigued by the high incidence of this brain abnormality. This incidence, while still remarkable, is lower than the 43% incidentally found in Wistar rats from two different vendors, and described in a previous study (Tu et al., 2014). As compared to said study, the degree of ventricular enlargement was higher in the rats we evaluated, determining a more severe ventriculomegaly. However, this seems to be not unusual. A very recent report by Ferris et al. (2019) described a single two‐year‐old typically behaving rat that was found to have massive hydrocephalus, similar to the most severe cases we present. In said rat, spatial memory was found to be preserved. Similarly, severe hydrocephalus (with variable severity) has been recently reported in a genetic model of renal cystic disease (Shim et al., 2019). Therefore, such large hydrocephalus is not unique to our study. Moreover, a longitudinal evaluation of the volume of the ventricular system was not performed in our study. Therefore, we cannot comment on the progression of the hydrocephalus. However, based on the lack of the classic sign of a domed head, characteristic of neonatal hydrocephalus, we suggest that ventricular enlargement occurs after fusion of the cranial sutures.

Animal models of neonatal hydrocephalus and ventriculomegaly exist (McAllister 2012; Volpon Santos, da Silva Lopes, Machado, & Santos de Oliveira, 2019). In this work we report similar anatomical findings, such as paper‐thin cerebral cortex and greatly enlarged brain ventricular system, as an incidental finding, not induced by any genetic or mechanical process. Ventriculomegalic Wistar rats have also been demonstrated to harbor arteriovenous malformations and white matter diffusion abnormalities (Tu et al., 2014). It is surprising that animals with such morphological abnormalities can show gross behavior and an external phenotype that is indistinguishable from control animals. Aside from the implications in memory described herein, these structural abnormalities are a potential source of error for experimental procedures, such as stereotaxic implantation or lesions (Tu et al., 2017). As such, and given the relatively high incidence of spontaneous ventriculomegaly, we suggest that all animals are screened through imaging prior to procedures that could be affected by such brain abnormalities.

In addition to the enlarged ventricles, the hippocampus was displaced and atrophied. The hippocampus is able to represent temporal aspects of events, which highlights its participation in choice tasks that involve waiting (Ferbinteanu & Shapiro, 2003; Peters & Buchel, 2010). On the other hand, clinical and experimental reports have also demonstrated that hippocampal damage directly impacts upon particular behaviors such as deciding between immediate and delayed rewards (Cheung & Cardinal, 2005; Gleichgerrcht et al., 2010). Therefore, based on this link, we decided to evaluate for the first time the effects of spontaneous ventriculomegaly in young adult rats when submitted to a couple of tasks where the hippocampus is crucial for creating long‐term retention memories while decision making.

Spontaneous alternation is a measure of exploratory behavior and spatial memory commonly evaluated in rodents (for review, see Dudchenko, 2004). The animals usually alternate, showing a tendency to explore the new environmental stimuli rather than re‐visiting the previously chosen path. Previous studies have reported that alternation tests can be performed in two manners, the free test and the forced test. Based on the latter one (Deacon & Rawlins, 2006; Lalonde, 2002), we blocked one of the arms in order to favor alternation behavior and a positive reinforcer was placed inside of the arms just to reward the alternation behavior. In general, alternation tests are usually used to evaluate the behavioral effects of lesions of the hippocampus, thalamus, basal ganglia, and prefrontal cortex (Bats et al., 2001; Dudchenko, Wood, & Eichenbaum, 2000; Kim & Frank, 2009; Lalonde, 2002). Moreover, when varying the length of the retention interval, the strength of the spatial working memory can also be estimated (Lalonde 2002). Our results show that rats with ventriculomegaly required 45% more training sessions to reach the requested accuracy value (80%) (Figure 3a) and, as expected, the alternation rates of both groups decreased at longer retention intervals, being more pronounced in the lesion rats. Similar to those reports where rats have surgically or chemically induced hippocampal lesions (Johnson, Olton, Gage, & Jenko, 1977; Pacteau, Einon, & Sinden, 1989). In this sense, other studies have also shown that animals with a lesioned prefrontal cortex reach lower alternation rates than control subjects, suggesting a deficit in spatial working memory (Le Marec et al., 2002; Izaki, Takita, & Akema, 2008). It could be argued that alterations in reward mechanisms are involved in the higher number of sessions required by the ventriculomegaly group to reach the 80% criterion in comparison to the control group. If this was the case, then subjects likely would have eaten more to compensate for the deficits in reward associated with food. However, this seems unlikely since no significant differences were observed in the weight of the subjects along the experiment, indicating that food consumption was similar for both groups. Moreover, as described in the results section and in contrast to control animals, rats with ventriculomegaly did not eat the food pellet when they had the opportunity to do so. Similar cognitive impairments, in a simple discrimination task and a delay matching to position task, have been observed in marmosets with lateral ventriculomegaly and hippocampal atrophy (Sadoun, Strelnikov, Bonte, Fonte, & Girard, 2015). As well, in the present experiment cognitive alterations were observed in the object recognition task, which does not involve reward.

Novel object recognition represents a useful experimental paradigm for studying the neural mechanisms of learning and memory in rodents (Ennaceur & Delacour, 1988). In general, the recognition process is considered to be integrated by the judgment of familiarity of items and the recollection of contextual (spatial and/or temporal) information where items were encountered (Brown & Aggleton, 2001). Experimental studies have reported that hippocampal damage can disrupt the ability to recall or express information about context (Balderas et al., 2008; Sutherland & McDonald, 1990). Specifically, the object‐in‐context recognition test is based on the association of a specific object (the stimulus) with a context (the place where it occurs). In this study, objects and contexts were already familiar to the animals, challenging them to correlate the new information (the “what” with the “where”). In particular, contextual recognition memory has been linked to a network of temporal regions that include the hippocampus (Malkova & Mishkin, 2003; Mumby, Tremblay, Lecluse, & Lehmann, 2005). Moreover, hippocampal‐specific protein synthesis is necessary to consolidate such information (Rossato et al., 2007). Similar to previous studies where permanent hippocampal lesions have deteriorated the animals’ ability to remember familiar objects in novel locations (Mumby et al., 2002), our findings clearly indicate hippocampal dysfunction related to spontaneous ventriculomegaly that translate into poor object‐in‐context recognition and memory consolidation. In addition to hippocampus, lateral entorhinal cortex has also been associated with episodic‐like memories in rodents, being crucial for integrating spatial and contextual information about objects (Wilson, Watanabe, Milner, & Ainge, 2013). In this sense, diverse studies have demonstrated that lateral entorhinal cortex neurons encode objects in the environment and the locations where objects were previously experienced, generating an episode retrieval (Tsao, Moser, & Moser, 2013; Tsao et al., 2018).

Since the installation in our Institute of a 7 Tesla MRI scanner in 2015, hundreds of animals (including mice, rats, and prairie voles) have been evaluated. Animals with such ventricular enlargement had never been seen before, which motivated our curiosity but at the same time alerted us. Therefore, we contacted the Institute's animal facility authorities shortly after our first observations of animals with hydrocephalus. The first measure taken was to replace the male rats used for reproduction with newly purchased males (Charles River Laboratories). A few weeks later, the frequency in which we identified animals with this lesion steeply declined. While certainly the right decision, this precluded the examination of more animals for further studies. We continue to monitor animal production with the aim to ensure that further experiments from our group and others are carried out in animals with normal ventricular systems.

## 5 CONCLUSION

The ventricular enlargement observed in the lesion animals was not induced. The magnitude directly affected the cortical thickness and morphology of several brain regions, impairing the animals’ decision‐making behaviors. Interestingly, despite the remarkable cerebral lesion observed in the animals, they did not display external phenotypic abnormalities, were able to develop normal behaviors and motor skills, and tried to decode the challenges when exposed to the memory tasks. Probably, considerable plasticity and compensation occurs; this will require further studies.

Considering that ventriculomegalic animals lack external phenotypic abnormalities (such as domed head or decreased body growth) nor develop atypical behaviors or motor skills, we have suggested to the Institute's scientific community to perform screening of their animals through a brief baseline MRI examination prior to any experimental procedure. Our lab has already established a 3‐min scanning protocol to avoid this uncertainty. Through our work, we extend this recommendation to the scientific community in general.

## CONFLICT OF INTEREST

None declared.

## AUTHOR CONTRIBUTION

HLM, DGM, and LC made substantial contributions to conception and design. HLM, DGM, and LMB made substantial contributions to acquisition of data. HLM, DGM, and LC made substantial contributions to analysis and interpretation of data. HLM and LC were involved in drafting the manuscript and revising it critically.

## Data Availability

The data that support the findings of this study are available from the corresponding author upon reasonable request.

## References

[brb31711-bib-0001] Balderas, I. , Rodriguez‐Ortiz, C. J. , Salgado‐Tonda, P. , Chavez‐Hurtado, J. , McGaugh, J. L. , & Bermudez‐Rattoni, F. (2008). The consolidation of object and context recognition memory involve different regions of the temporal lobe. Learning & Memory, 15(9), 618–624. 10.1101/lm.1028008 18723431 PMC2632790

[brb31711-bib-0002] Bats, S. , Thoumas, J. L. , Lordi, B. , Tonon, M. C. , Lalonde, R. , & Caston, J. (2001). The effects of a mild stressor on spontaneous alternation in mice. Behavioural Brain Research, 118(1), 11–15. 10.1016/s0166-4328(00)00285-0 11163629

[brb31711-bib-0003] Brown, M. W. , & Aggleton, J. P. (2001). Recognition memory: What are the roles of the perirhinal cortex and hippocampus? Nature Reviews Neuroscience, 2(1), 51–61. 10.1038/35049064 11253359

[brb31711-bib-0004] Cheung, T. H. , & Cardinal, R. N. (2005). Hippocampal lesions facilitate instrumental learning with delayed reinforcement but induce impulsive choice in rats. BMC Neuroscience, 6, 36 10.1186/1471-2202-6-36 15892889 PMC1156904

[brb31711-bib-0005] Deacon, R. M. , & Rawlins, J. N. (2006). T‐maze alternation in the rodent. Nature Protocols, 1(1), 7–12. 10.1038/nprot.2006.2 17406205

[brb31711-bib-0006] del Bigio, M. R. (2010). Neuropathology and structural changes in hydrocephalus. Developmental Disabilities Research Reviews, 16(1), 16–22. 10.1002/ddrr.94 20419767

[brb31711-bib-0007] Dix, S. L. , & Aggleton, J. P. (1999). Extending the spontaneous preference test of recognition: Evidence of object‐location and object‐context recognition. Behavioural Brain Research, 99(2), 191–200. 10.1016/S0166-4328(98)00079-5 10512585

[brb31711-bib-0008] Dudchenko, P. A. (2004). An overview of the tasks used to test working memory in rodents. Neuroscience and Biobehavioral Reviews, 28(7), 699–709. 10.1016/j.neubiorev.2004.09.002 15555679

[brb31711-bib-0009] Dudchenko, P. A. , Wood, E. R. , & Eichenbaum, H. (2000). Neurotoxic hippocampal lesions have no effect on odor span and little effect on odor recognition memory but produce significant impairments on spatial span, recognition, and alternation. The Journal of Neuroscience, 20(8), 2964–2977. 10.1523/JNEUROSCI.20-08-02964.2000 10751449 PMC6772220

[brb31711-bib-0010] Engelsen, B. A. , Fosse, V. M. , Myrseth, E. , & Fonnum, F. (1985). Elevated concentrations of glutamate and aspartate in human ventricular cerebrospinal fluid (vCSF) during episodes of increased CSF pressure and clinical signs of impaired brain circulation. Neuroscience Letters, 62(1), 97–102. 10.1016/0304-3940(85)90290-3 2866476

[brb31711-bib-0011] Ennaceur, A. , & Delacour, J. (1988). A new one‐trial test for neurobiological studies of memory in rats. 1: Behavioral data. Behavioural Brain Research, 31(1), 47–59. 10.1016/0166-4328(88)90157-x 3228475

[brb31711-bib-0012] Fellows, B. J. (1967). Chance stimulus sequences for discrimination tasks. Psychological Bulletin, 67(2), 87–92. 6045339 10.1037/h0024098

[brb31711-bib-0013] Ferbinteanu, J. , & Shapiro, M. L. (2003). Prospective and retrospective memory coding in the hippocampus. Neuron, 40(6), 1227–1239. 10.1016/S0896-6273(03)00752-9 14687555

[brb31711-bib-0014] Ferris, C. F. , Cai, X. , Qiao, J. , Switzer, B. , Baun, J. , Morrison, T. , … Kulkarni, P. (2019). Life without a brain: Neuroradiological and behavioral evidence of neuroplasticity necessary to sustain brain function in the face of severe hydrocephalus. Scientific Reports, 9(1), 16479 10.1038/s41598-019-53042-3 31712649 PMC6848215

[brb31711-bib-0015] Floresco, S. B. , Seamans, J. K. , & Phillips, A. G. (1997). Selective roles for hippocampal, prefrontal cortical, and ventral striatal circuits in radial‐arm maze tasks with or without a delay. The Journal of Neuroscience, 17(5), 1880–1890. 10.1523/JNEUROSCI.17-05-01880.1997 9030646 PMC6573377

[brb31711-bib-0016] Gleichgerrcht, E. , Ibañez, A. , Roca, M. , Torralva, T. , & Manes, F. (2010). Decision‐making cognition in neurodegenerative diseases. Nature Reviews Neurology, 6(11), 611–623. 10.1038/nrneurol.2010.148 21045795

[brb31711-bib-0017] Golomb, J. , de Leon, M. J. , George, A. E. , Kluger, A. , Convit, A. , Rusinek, H. , … Ferris, S. H. (1994). Hippocampal atrophy correlates with severe cognitive impairment in elderly patients with suspected normal pressure hydrocephalus. Journal of Neurology, Neurosurgery, and Psychiatry, 57, 590–593. 10.1136/jnnp.57.5.590 PMC1072921 8201330

[brb31711-bib-0018] Götz, J. , Bodea, L. G. , & Goedert, M. (2018). Rodent models for Alzheimer disease. Nature Reviews Neuroscience, 19(10), 583–598. 10.1038/s41583-018-0054-8 30194347

[brb31711-bib-0019] Gupta, R. , Duff, M. C. , Denburg, N. L. , Cohen, N. J. , Bechara, A. , & Tranel, D. (2009). Declarative memory is critical for sustained advantageous complex decision‐making. Neuropsychologia, 47(7), 1686–1693. 10.1016/j.neuropsychologia.2009.02.007 19397863 PMC2697903

[brb31711-bib-0020] Hölscher, C. (2003). Time, space and hippocampal functions. Reviews in the Neurosciences, 14(3), 253–284. 10.1515/REVNEURO.2003.14.3.253 14513868

[brb31711-bib-0021] Izaki, Y. , Takita, M. , & Akema, T. (2008). Specific role of the posterior dorsal hippocampus‐prefrontal cortex in short‐term working memory. The European Journal of Neuroscience, 27(11), 3029–3034. 10.1111/j.1460-9568.2008.06284.x 18540879

[brb31711-bib-0022] Johnson, C. T. , Olton, D. S. , Gage 3rd, F. H. , & Jenko, P. G. (1977). Damage to hippocampus and hippocampal connections: Effects on DRL and spontaneous alternation. Journal of Comparative and Physiological Psychology, 91(3), 508–522. 10.1037/h0077346 874119

[brb31711-bib-0023] Kim, S. M. , & Frank, L. M. (2009). Hippocampal lesions impair rapid learning of a continuous spatial alternation task. PLoS One, 4(5), e5494 10.1371/journal.pone.0005494 19424438 PMC2674562

[brb31711-bib-0024] Korzh, V. (2018). Development of brain ventricular system. Cellular and Molecular Life Sciences, 75(3), 375–383. 10.1007/s00018-017-2605-y 28780589 PMC5765195

[brb31711-bib-0025] Lalonde, R. (2002). The neurobiological basis of spontaneous alternation. Neuroscience and Biobehavioral Reviews, 26(1), 91–104. 10.1016/S0149-7634(01)00041-0 11835987

[brb31711-bib-0026] Le Marec, N. , Ethier, K. , Rompre, P. P. , & Godbout, R. (2002). Involvement of the medial prefrontal cortex in two alternation tasks using different environments. Brain and Cognition, 48(2–3), 432–436. 12030483

[brb31711-bib-0027] Lindquist, B. , Persson, E. K. , Uvebrant, P. , & Carlsson, G. (2008). Learning, memory and executive functions in children with hydrocephalus. Acta Paediatrica (Oslo, Norway:1992), 97, 596–601. 10.1111/j.1651-2227.2008.00747.x 18394105

[brb31711-bib-0028] Lopes, L. S. , Slobodian, I. , & del Bigio, M. R. (2009). Characterization of juvenile and young adult mice following induction of hydrocephalus with kaolin. Experimental Neurology, 219(1), 187–196. 10.1016/j.expneurol.2009.05.015 19460371

[brb31711-bib-0029] Malkova, L. , & Mishkin, M. (2003). One‐trial memory for object‐place associations after separate lesions of hippocampus and posterior parahippocampal region in the monkey. The Journal of Neuroscience, 23(5), 1956–1965. 10.1523/JNEUROSCI.23-05-01956.2003 12629201 PMC6741967

[brb31711-bib-0030] McAllister 2nd, J. P. (2012). Pathophysiology of congenital and neonatal hydrocephalus. Seminars in Fetal & Neonatal Medicine, 17(5), 285–294. 10.1016/j.siny.2012.06.004 22800608

[brb31711-bib-0031] Metzger, J. M. , & Emborg, M. E. (2019). Autonomic dysfunction in Parkinson disease and animal models. Clinical Autonomic Research, 29(4), 397–414. 10.1007/s10286-018-005847 30604165 PMC6606399

[brb31711-bib-0032] Mizoguchi, K. , Yuzurihara, M. , Ishige, A. , Sasaki, H. , Chui, D. H. , & Tabira, T. (2000). Chronic stress induces impairment of spatial working memory because of prefrontal dopaminergic dysfunction. The Journal of Neuroscience, 20(4), 1568–1574. 10.1523/JNEUROSCI.20-04-01568.2000 10662846 PMC6772382

[brb31711-bib-0033] Mori, K. , Shimada, J. , Kurisaka, M. , Sato, K. , & Watanabe, K. (1995). Classification of hydrocephalus and outcome treatment. Brain & Development, 17(5), 338–348. 8579221 10.1016/0387-7604(95)00070-r

[brb31711-bib-0034] Mumby, D. G. , Gaskin, S. , Glenn, M. J. , Schramek, T. E. , & Lehmann, H. (2002). Hippocampal damage and exploratory preferences in rats: Memory for objects, places, and contexts. Learning & Memory, 9(2), 49–57. 10.1101/lm.41302 11992015 PMC155935

[brb31711-bib-0035] Mumby, D. G. , Tremblay, A. , Lecluse, V. , & Lehmann, H. (2005). Hippocampal damage and anterograde object‐recognition in rats after long retention intervals. Hippocampus, 15(8), 1050–1056. 10.1002/hipo.20122 16145694

[brb31711-bib-0036] Nirwan, N. , Vyas, P. , & Vohora, D. (2018). Animal models of status epilepticus and temporal lobe epilepsy: A narrative review. Reviews in the Neurosciences, 29(7), 757–770. 10.1515/revneuro-2017-0086 29565791

[brb31711-bib-0037] Pacteau, C. , Einon, D. , & Sinden, J. (1989). Early rearing environment and dorsal hippocampal ibotenic acid lesions: Long‐term influences on spatial learning and alternation in the rat. Behavioural Brain Research, 34(1–2), 79–96. 2765174 10.1016/s0166-4328(89)80092-0

[brb31711-bib-0038] Peters, J. , & Buchel, C. (2010). Episodic future thinking reduces reward delay discounting through an enhancement of prefrontal‐mediotemporal interactions. Neuron, 66(1), 138–148. 10.1016/j.neuron.2010.03.026 20399735

[brb31711-bib-0039] Peterson, K. A. , Mole, T. B. , Keong, N. C. H. , DeVito, E. E. , Savulich, G. , Pickard, J. D. , & Sahakian, B. J. (2018). Structural correlates of cognitive impairment in normal pressure hydrocephalus. Acta Neurologica Scandinavica, 139(3), 305–312. 10.1111/ane.13052 30428124 PMC6492129

[brb31711-bib-0040] Rossato, J. I. , Bevilaqua, L. R. , Myskiw, J. C. , Medina, J. H. , Izquierdo, I. , & Cammarota, M. (2007). On the role of hippocampal protein synthesis in the consolidation and reconsolidation of object recognition memory. Learning & Memory, 14(1), 36–46. 10.1101/lm.422607 17272651 PMC1838544

[brb31711-bib-0041] Sadoun, A. , Strelnikov, K. , Bonte, E. , Fonte, C. , & Girard, P. (2015). Cognitive impairment in a young marmoset reveals lateral ventriculomegaly and a mild hippocampal atrophy: A case report. Scientific Reports, 5, 16046 10.1038/srep16046 26527211 PMC4630607

[brb31711-bib-0042] Sasaki, S. , Goto, H. , Nagano, H. , Furuya, K. , Omata, Y. , Kanazawa, K. , … Collmann, H. (1983). Congenital hydrocephalus revealed in the inbred rat LEW/Jms. Neurosurgery, 13(5), 548–554. 10.1227/00006123-198311000-00011 6606138

[brb31711-bib-0043] Shim, J. W. , Territo, P. R. , Simpson, S. , Watson, J. C. , Jiang, L. , Riley, A. A. , … Blazer‐Yost, B. L. (2019). Hydrocephalus in a rat model of Meckel Gruber syndrome with a TMEM67 mutation. Scientific Reports, 9(1), 1069 10.1038/s41598-018-37620-5 30705305 PMC6355840

[brb31711-bib-0044] Spanswick, S. C. , & Sutherland, R. J. (2010). Object/context‐specific memory deficits associated with loss of hippocampal granule cells after adrenalectomy in rats. Learning & Memory, 17(5), 241–245. 10.1101/lm.1746710 20410060 PMC2893217

[brb31711-bib-0045] Sutherland, R. J. , & McDonald, R. J. (1990). Hippocampus, amygdala, and memory deficits in rats. Behavioural Brain Research, 37(1), 57–79. 10.1016/0166-4328(90)90072-m 2310495

[brb31711-bib-0046] Tsao, A. , Moser, M. B. , & Moser, E. I. (2013). Traces of experience in the lateral entorhinal cortex. Current Biology, 23(5), 399–405. 10.1016/j.cub.2013.01.036 23434282

[brb31711-bib-0047] Tsao, A. , Sugar, J. , Lu, L. , Wang, C. , Knierim, J. J. , Moser, M. B. , & Moser, E. I. (2018). Integrating time from experience in the lateral entorhinal cortex. Nature, 561(7721), 57–62. 10.1038/s41586-018-0459-6 30158699

[brb31711-bib-0048] Tu, T. W. , Lescher, J. D. , Williams, R. A. , Jikaria, N. , Turtzo, L. C. , & Frank, J. A. (2017). Abnormal injury response in spontaneous mild ventriculomegaly Wistar rat brains: A pathological correlation study of diffusion tensor and magnetization transfer imaging in mild traumatic brain injury. Journal of Neurotrauma, 34(1), 248–256. 10.1089/neu.2015.4355 26905805 PMC5198143

[brb31711-bib-0049] Tu, T. W. , Turtzo, L. C. , Williams, R. A. , Lescher, J. D. , Dean, D. D. , & Frank, J. A. (2014). Imaging of spontaneous ventriculomegaly and vascular malformations in Wistar rats: Implications for preclinical research. Journal of Neuropathology and Experimental Neurology, 73(12), 1152–1165. 10.1097/NEN.0000000000000140 25383642 PMC4232989

[brb31711-bib-0050] Tully, H. M. , & Dobyns, W. B. (2014). Infantile hydrocephalus: A review of epidemiology, classification and causes. European Journal of Medical Genetics, 57(8), 359–368. 10.1016/j.ejmg.2014.06.002 24932902 PMC4334358

[brb31711-bib-0051] Volpon Santos, M. , da Silva Lopes, L. , Machado, H. R. , & Santos de Oliveira, R. (2019). Behavioral and biochemical features of the course and surgical treatment of experimental obstructive hydrocephalus in young rats. Developmental Neuroscience, 18, 1–10. 10.1159/000497433 30999305

[brb31711-bib-0052] Wang, D. C. , Liu, P. C. , Hung, H. S. , & Chen, T. J. (2014). Both PKMζ and KIBRA are closely related to reference memory but not working memory in a T‐maze task in rats. Journal of Comparative Physiology. A Neuroethology, Sensory, Neural, and Behavioral Physiology, 200(1), 77–82. 10.1007/s00359-013-0862-2 24141945

[brb31711-bib-0053] Wilson, D. I. G. , Watanabe, S. , Milner, H. , & Ainge, J. A. (2013). Lateral entorhinal cortex is necessary for associative but not nonassociative recognition memory. Hippocampus, 23(12), 1280–1290. 10.1002/hipo.22165 23836525 PMC4030623

[brb31711-bib-0054] Yushkevich, P. A. , Piven, J. , Hazlett, H. C. , Smith, R. G. , Ho, S. , Gee, J. C. , & Gerig, G. (2006). User‐guided 3D active contour segmentation of anatomical structures: Significantly improved efficiency and reliability. NeuroImage, 31(3), 1116–1128. 10.1016/j.neuroimage.2006.01.015 16545965

[brb31711-bib-0055] Zhang, J. , Williams, M. A. , & Rigamonti, D. (2006). Genetics of human hydrocephalus. Journal of Neurology, 253(10), 1255–1266. 10.1007/s00415-006-0245-5 16773266 PMC1705504

[brb31711-bib-0056] Zielinska, D. , Rajtar‐Zembaty, A. , & Starowicz‐Filip, A. (2017). Cognitive disorders in children’s hydrocephalus. Neurologia I Neurochirurgia Polska, 51(3), 234–239. 10.1016/j.pjnns.2017.02.001 28284447

